# Diversity of nasal microbiota and its interaction with surface microbiota among residents in healthcare institutes

**DOI:** 10.1038/s41598-019-42548-5

**Published:** 2019-04-16

**Authors:** Chang-Hua Chen, Ming-Li Liou, Cheng-Yang Lee, Ming-Chuan Chang, Han-Yueh Kuo, Tzu-Hao Chang

**Affiliations:** 1 Division of Infectious Diseases, Department of Internal Medicine, Changhua Christian Hospital, Changhua City, Taiwan; 2 Department of Nursing, College of Nursing, Hung Kuang University, Taichung County, Taiwan; 3 Ph.D. Program in Translational Medicine, National Chung Hsing University, Taichung City, Taiwan; 4 Rong Hsing Research Center For Translational Medicine, National Chung Hsing University, Taichung City, Taiwan; 5 Department of Medical Laboratory Science and Biotechnology, Yuanpei University, Hsin-Chu City, Taiwan; 6 Graduate Institute of Biomedical Informatics, Taipei Medical University, Taipei City, Taiwan; 7 Department of Medicine, Nantou Christian Hospital, Nantou City, Taiwan; 8 Department of Medicine, National Taiwan University Hospital Hsin-Chu Branch, Hsin-Chu City, Taiwan; 9 Clinical Big Data Research Center, Taipei Medical University Hospital, Taipei City, Taiwan; 10 School of Medicine, National Taiwan University, Taipei City, Taiwan

## Abstract

Nasal microbial communities may have crucial implications for human health, including for residents of healthcare institutes (HCIs). Factors that determine the diversity of nasal microbiota in HCIs remain unclear. Herein, we used 16S rRNA amplicon sequencing to investigate the relationship between nasal and surface microbiota in three HCIs. Participants were classified into a hospitalised or nonhospitalised group based on their most recent date of hospitalisation. A total of 88 nasal samples and 83 surface samples were analysed. *Dysgonomonas* and *Corynebacterium* were the most abundant taxa in the surface and nasal samples, respectively. Significant differences were discovered in microbiota diversity among HCIs when comparing the surface and nasal samples. Fifteen taxa were identified as present in all the surface and nasal samples. SourceTracker analysis revealed that the ventilation conditions of environment might be associated with the proportion of shared microbial communities between nasal and surface. Additionally, as compared with the nonhospitalised group, the hospitalised group had a higher proportion of surface microbiota in their nasal samples, which might lead to a higher risk of human-related microorganisms or pathogens colonising the nasal cavity. The data suggest that nasal bacterial diversity could be influenced by both health status and living environment. Our results therefore highlight the importance of the indoor environment for HCI residents.

## Introduction

Humans body hosts diverse microbiota^1^. Dispersal of microbial communities between humans and the built environment can occur through directly surface or airborne release, and transmit pathogens to other individuals and to indoor surfaces^2^. The global trend towards increased longevity has placed new emphasis on the healthcare environment. The composition and diversity of surface microbiota found in healthcare institutes (HCIs) are affected by several factors such as cleaning processes used in the institutions^3^, the disinfection of indoor air^4–6^, and the source of ventilated air^5^.

Culture-independent, high-throughput molecular sequencing approaches based on the polymerase chain reaction(PCR) amplification and sequencing of genes encoding the small subunit ribosomal RNA (16S rRNA)^7^ have transformed the study of microbial diversity^5^, and it has led to an expansion of knowledge regarding the microbial communities across built environments. Recent studies showed that human’s microbiota potentially impacts environmental microbiota. For example, Leung *et al*. suggested that occupants of the building may have significant effects on their environment^8^, and Lax *et al*.’s study reported environmental microbiota be rapidly colonized by the human’s microbiota^9^. Additionally, Lai *et al*. also revealed that human’s microbiome could be attributed from environmental microbiome^10^.

Richly diverse commensal microbiota are crucial for sustaining an equilibrium in the bacterial community, maintaining the integrity of the mucosal barrier^1,11–14^, and many other aspects of health, such as resistance to infection^15^, an effective immune system^16,17^, and favourable nutritional status^18^. A pronounced variability exists in an individual’s microbiota across body sites. Moreover, the interaction of the mucosal immune system with the inflammatory system can impose selective pressures, and certain microorganisms, including human-related microorganisms, can survive under stressful conditions that impair the fitness of most commensal microbes^19^. Diseases such as chronic rhinosinusitis^20^ can result from dysbiosis of nasal microbiota.

Among HCIs in Taiwan, a rapid increase in the number of long-term care facilities (LTCFs) has resulted in a focus on the association between environmental cleanliness and resident health. Residents of LTCFs may release their own unique microbial cloud^2^, and colonisation of the skin microbiome for residents of LTCFs is highly variable^21^. Bacterial outbreaks have been reported in LTCFs^22^ and may result in the dissemination of multiple-drug-resistant microorganisms from LTCFs to the community or to the wider healthcare delivery system through acute care hospitals in the area. In a prospective study, a high incidence of human-related microorganisms of the nasal carriage was discovered at LTCFs^23^. A few studies have also reported the presence of other human-related microorganisms in the nasal cavity^23–25^. Nowadays, there is still a lack of a comprehensive and detailed analysis of the relationship between nasal microbiota and environmental microbiota. It is essential to expand our knowledge of human nasal microbiota diversity and the function of the bacterial community in HCIs. Therefore, in this study, we used a 16S rRNA sequencing approach to survey surface microbiota in three HCIs and the nasal microbiota of their residents. Three main aspects were considered for analysis and discussion in this study: (1) understanding the composition of the nasal microbiota of individuals in HCIs with different environmental building conditions, (2) identifying core microbiota present in all HCIs and their residents, and (3) analysing how surface microbiota affect nasal microbiota or vice versa for each HCI and resident.

## Results

A total of 88 nasal samples were collected from participants, and 83 surface samples were collected from the three HCIs (NH, NC, and SC). After demultiplexing and quality control checking, a total of 2,002,198 sequence reads were obtained from the surface samples, with a median of 17,303 reads and a median read length of 237 bp per surface sample. A total of 2,648,842 sequence reads were obtained from the nasal samples, with a median of 21,822 reads and a median read length of 234 bp per nasal sample. After microbial taxonomy assignment, a mean of 23,589 operational taxonomic unit (OTU)-mapped reads and 1,433 OTUs were obtained per surface sample, and a mean of 21,086 OTU-mapped reads and 1,203 OTUs were obtained per nasal sample. The OTU at the genus level in both the surface and nasal samples is provided in Supplementary File 1.

### Microbial composition and diversity of surface and nasal microbiota

**Figure 1:**
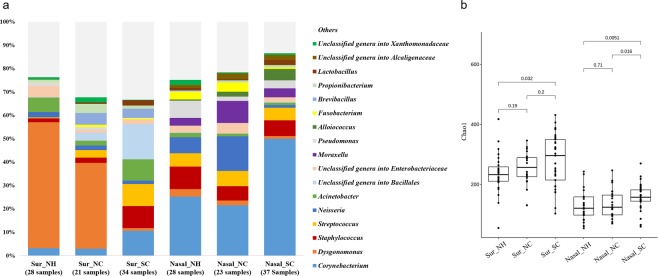
Microbiota distribution and diversity in surface and nasal samples within the three healthcare institutes (**a**) boxplot of relative proportion of bacterial taxa (**b**) boxplot of the Chao1 index value. Note: NH, NC, and SC are the three healthcare institutes. Abbreviations: Sur: surface microbiota; Nasal: nasal microbiota.

Figure 1a displays the microbiota distribution in the surface samples from the three HCIs as well as in the nasal samples of their residents; the composition of microbial communities in each sample is presented in Fig. S1. As shown in Fig. 1a, the major microbial taxa in the surface samples were *Corynebacterium* (abundance range: 2.88–10.43%), *Dysgonomonas* (1.20–53.62%), *Acinetobacter* (1.98–8.95%), *Neisseria* (1.41–2.28%), *Staphylococcus* (1.63–9.68%), and unclassified genera into *Bacillales* (0.21–14.73%). The major microbes in the nasal communities were *Corynebacterium* (21.53–48.60%), *Neisseria* (1.11–14.80%), *Staphylococcus* (6.12–9.61%), and *Streptococcus* (5.18–6.47%). The median relative abundance of these major microbial communities are shown in Table S1.

The Chao1 diversity of surface microbiota was higher than that of nasal microbiota (Fig. 1b). A significant difference was also discovered between NH and SC in both the surface and nasal samples, with *p* values of 0.032 and 0.0051, respectively. With respect to surface microbial communities, *Dysgonomonas* was more abundant at NH and NC than at SC (*p* < 0.001; Fig. S2). Additionally, *Corynebacterium* was more abundant at SC than at NH and NC (*p* < 0.001) with respect to nasal microbial communities.

**Figure 2:**
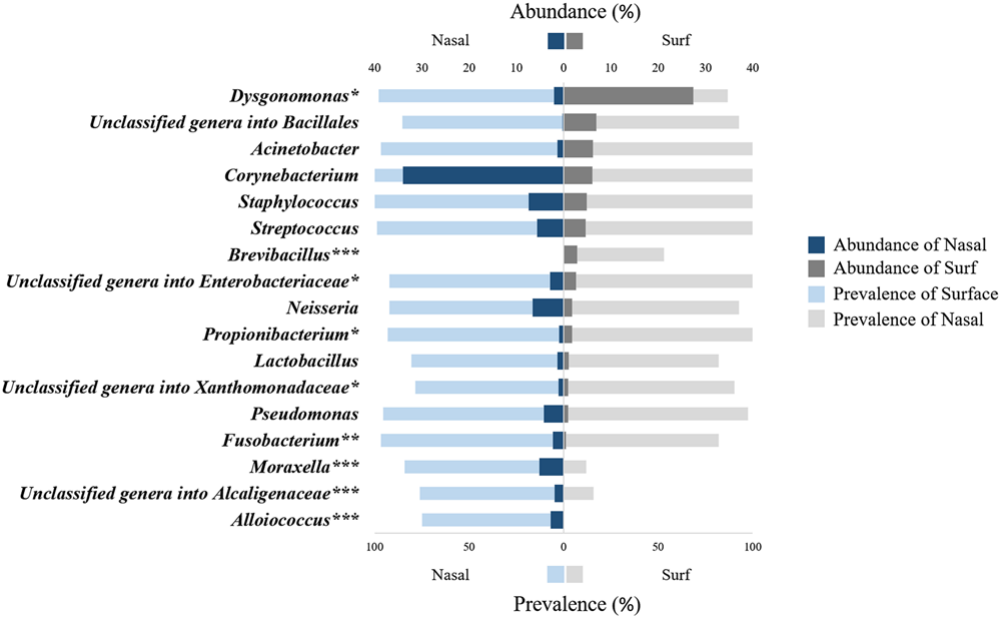
Relatively abundant taxa and their prevalence in surface and nasal samples. Note: Only taxa with >1% average abundance are displayed. NH, NC, and SC are the healthcare institutes. Abbreviations: Sur_Abundance: abundance of taxa in the surface microbiota; Sur_Prevalence: prevalence of taxa in the surface microbiota; Nasal _Abundance: abundance of taxa in the nasal microbiota; Nasal _Prevalence: prevalence of taxa in the nasal microbiota.

Figure 2 indicates the taxon with an average proportion of more than 1% in all samples and their prevalence. With the exception of *Brevibacillus* and *Alloiococcus*, the taxon showed a prevalence between 12% and 100% across the two communities. *Dysgonomonas*, *Corynebacterium*, *Staphylococcus*, *Streptococcus* and unclassified genera into *Enterobacteriaceae* were not only with highly prevalent (>80%) but also with relatively abundant (>2%) in both nasal and surface microbial communities.

**Figure 3:**
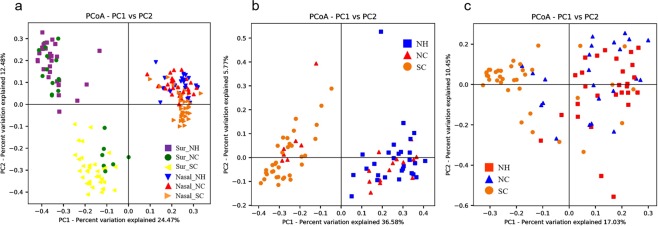
Principal component analysis of bacterial communities in (**a**) the surface and nasal samples (**b**) the surface samples and (**c**) the nasal samples obtained from the three healthcare institutes. Note: NH, NC, and SC are the three healthcare institutes. Abbreviations: Sur: surface microbiota; Nasal: nasal microbiota.

Principle component analysis was performed using a Bray-Curtis distance (BC distance) matrix to determine relationships among various bacterial communities in the three HCIs. The principal component analysis (PCoA) plot in Fig. 3 shows differences between surface and nasal microbiota but also reveals a notable difference between the HCIs (NH and NC versus SC). The result of the analysis of similarities (ANOSIM) test indicates that surface microbiota at SC formed a cluster distinct from the clusters from NH (R = 0.89, *p* < 0.001) and NC (R = 0.062, *p* < 0.001). Of nasal microbiota from residents of the three HCIs, SC was again significantly different from NH (R = 0.39, *p* < 0.001) and NC (R = 0.37, *p* < 0.001). Additionally, the analysis of inter-group distances was depicted in Fig. S3 using BC distance. Results showed wider variations of inter-group distances in surface groups as compared to nasal groups. Surface bacterial communities showed lower distances between NH and NC, and a little higher distances between NH and SC as compared to others. Nasal bacterial communities possessed relatively similar distances among all locations.

### Core microbiota and human-associated microorganisms in surface and nasal samples

**Figure 4:**
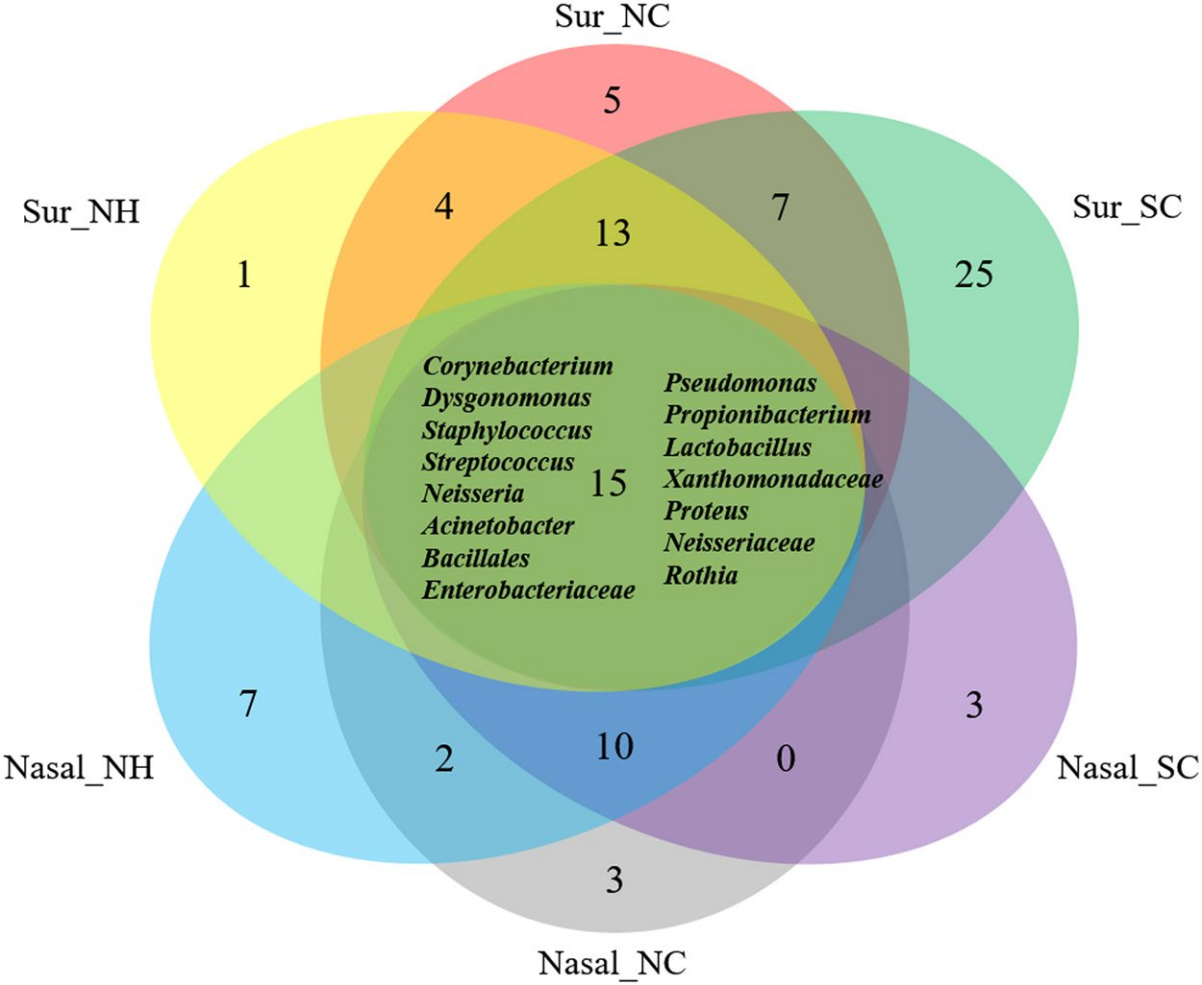
Taxa in both surface and nasal samples from the three healthcare institutes. Note: Taxa with a proportion of >0.1% in any one of the six sets in the Venn diagram were collected for analysis. Note: NH, NC, and SC are the three healthcare institutes. Abbreviations: Sur: surface microbiota; Nasal: nasal microbiota.

Figure 4 shows the number of taxa shared among the surface and nasal samples from the three HCIs with more than 0.1% average abundance. The detailed proportions of read counts for all the intersecting taxa are listed in Supplementary File 2. The data show that 15 taxa (core microbiota; including *Corynebacterium*, *Dysgonomonas*, *Staphylococcus*, *Streptococcus*, *Acinetobacter*, unclassified genera into *Bacillales*, unclassified genera into *Enterobacteriaceae*, *Pseudomonas*, *Propionibacterium*, *Lactobacillus*, unclassified genera into *Xanthomonadaceae*, *Proteus*, unclassified genera into *Neisseriaceae*, and *Rothia*) were shared between surface and nasal microbiota and that these taxa represent more than 67% of all sequences.

A more detailed investigation of core microbiota is presented in Table 1. Although the 15 taxa were present in most samples, their abundance across the groups differed considerably, ranging from 0.11% to 53%. *Staphylococcus*, *Pseudomonas*, Unclassified genera into *Neisseriaceae*, and *Rothia*, showed no significant difference of abundance between surface and nasal bacterial communities. Numerous taxa had the highest proportions in both surface and nasal bacterial communities. For example, *Dysgonomonas* not only had the highest proportion in surface bacterial communities from NH (53.62%) as compared with it from NC (35.07%) and SC (1.2%), but also possessed the highest proportion (3.16%) in nasal bacterial communities from NH as compared with it from NC (2.01%) and SC (1.15%). Similarly, *Pseudomonas* also showed the highest proportion in surface (1.58%) and nasal (1.25%) bacterial community from NH. Additionally, unclassified genera into *Neisseriaceae* had the highest proportion in both surface (0.28%) and nasal (1.36%) bacterial communities from NC. *Corynebacterium* (10.43% in surface and 48.60% in nasal), *Lactobacillus* (2.23% and 2.13%), and unclassified genera into *Bacillales* (14.73% and 0.29%) had the highest proportions in both bacterial communities from SC.

**Table 1:** Fifteen core microbiomes shared among samples and nasal bacterial communities from three healthcare institutes. Note: NH, NC, and SC are the three healthcare institutes. Abbreviations: Sur: surface microbiota; Nasal: nasal microbiota.

Taxonomy	Relative abundance (mean) (%)						Statistical comparisons		
	Sur_NH	Sur_NC	Sur_SC	Nasal_NH	Nasal_NC	Nasal_SC	Surface vs Nasal (p-value^a^)	Among Surfaces (p-value^b^)	Among Nasals (p-value^b^)
Corynebacterium** *	3.21	2.88	**10.43**	25.1	21.53	**48.6**	**5.02E-19**	**4.21E-08**	**2.39E-05**
Dysgonomonas**	**53.62**	35.07	1.2	**3.16**	2.01	1.15	**2.51E-04**	**9.85E-10**	**2.95E-06**
*Staphylococcus*	1.63	2.24	9.18	9.61	6.12	6.59	2.67E-01	**4.54E-07**	3.95E-01
*Streptococcus* *	0.41	3	9.25	5.6	6.47	5.18	**1.63E-03**	**8.59E-07**	6.13E-01
*Neisseria* *	2.28	1.87	1.41	6.84	14.8	1.11	**4.98E-02**	8.89E-02	**7.14E-03**
Acinetobacter** *	6.17	1.98	8.95	1.81	1.07	0.98	**3.13E-15**	1.16E-01	7.98E-01
Unclassified genera into Bacillales** *	0.21	3.34	**14.73**	0.24	0.16	**0.29**	**3.18E-08**	**1.56E-10**	**1.78E-05**
*Unclassified genera into Enterobacteriaceae* *	4.7	1.04	1.98	2.79	4.42	1.92	**3.51E-03**	**1.58E-02**	9.63E-01
*Pseudomonas*	**1.58**	1.14	0.22	**7.25**	1.88	3.33	7.66E-01	**1.07E-08**	2.50E-01
Propionibacterium** *	1.01	3.73	1.27	0.73	0.97	1.03	**1.95E-06**	1.16E-01	**1.87E-06**
*Lactobacillus* *	0.12	0.63	**2.23**	1.1	0.35	**2.13**	**3.14E-02**	**3.04E-05**	1.80E-01
Unclassified genera into Xanthomonadaceae**	0.99	2.07	0.15	2.1	0.43	0.71	**3.03E-04**	**2.78E-07**	2.86E-01
Proteus**	1.58	0.4	0.2	0.38	2.16	0.35	**7.87E-04**	**1.35E-06**	7.52E-02
*Unclassified genera into Neisseriaceae*	0.19	**0.28**	0.28	0.74	**1.36**	0.43	5.83E-01	2.09E-01	8.55E-02
*Rothia*	0.12	0.12	0.14	0.29	1.23	0.11	9.42E-01	8.82E-02	5.80E-01

* < 0.05; ** < 0.001; *** < 0.0001 for the taxon with significantly different abundance.

^a^Mann-Whitney U test; ^b^Kruskal-Wallis test.

Among all the most prevalent microbiota types, nine taxa are human-related microorganisms, including *Staphylococcus*, *Streptococcus*, *Neisseria*, *Acinetobacter*, unclassified genera into *Enterobacteriaceae*, *Propionibacterium*, *Proteus*, and *Pseudomonas*. Comparing the HCIs, it is notable that *Dysgonomonas*, *Neisseria*, and *Proteus* were more abundant in both the surface and nasal samples from NH and NC, whereas *Corynebacterium*, unclassified genera into *Bacillales*, and *Lactobacillus* were more abundant in both the surface and nasal samples from SC. This suggests that a correlation may exist between surface and nasal microbiota.

### Correlation analysis between surface and nasal microbiota

**Figure 5:**
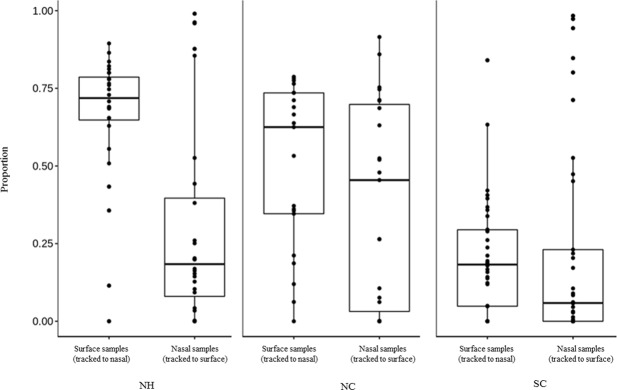
Boxplot of the proportion of nasal microbes from each sample tracked to surface and the proportion of surface microbes from surface samples tracked to nasal within different locations. Note: NH, NC, and SC are the three healthcare institutes.

Here, we applied a Bayesian-based approach, SourceTracker, to estimate the proportion of shared microbiota between the nasal sample and its corresponding environment. Figure 5 depicts the boxplot of the proportion of nasal microbes from each sample tracked to surfaces as a source and the proportion of surface microbes from surface samples tracked to nasal. The analysis results demonstrate that surface samples in NH and NC possess higher proportions of reads attributed from nasal samples as compared to that in SC. Notably, similar observations are also shown in nasal samples, which might be attributed to the different ventilation conditions, i.e. both NH and NC are with a central air conditioner, but SC is a window-ventilated site. Thus, nasal microbiota and surface microbiota shared fewer reads to each other in SC than that in NH and NC. The present results suggest that a bi-directional interaction might exist between nasal and surface bacterial communities.

**Figure 6:**
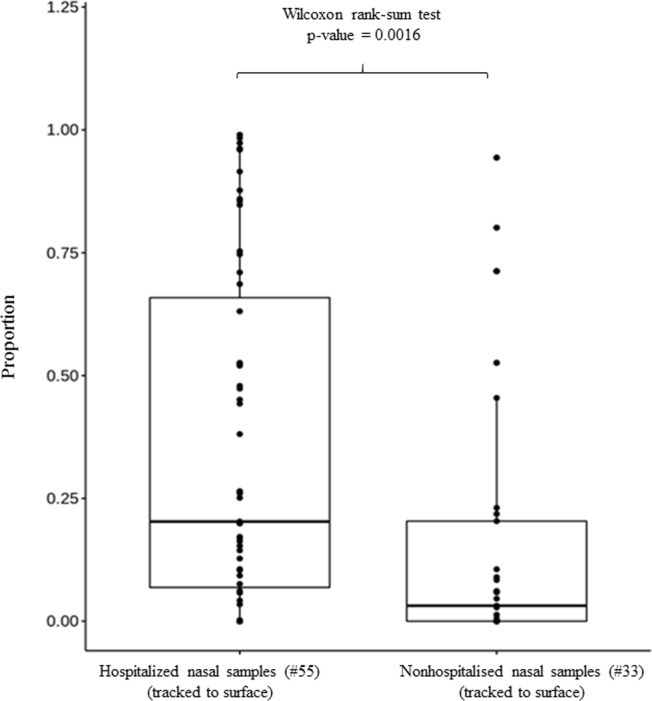
Boxplot of the proportion of nasal microbes from each sample in hospitalised and nonhospitalised groups tracked to surface.

To further analyse the effects of surface microbiota on nasal samples, we separated participants into a hospitalised group, composed of individuals who had been hospitalised within the 1 year prior to the study, and a nonhospitalised group, comprising individuals who had not been hospitalised within the 1 year prior to the study. As illustrated in Fig. 6, the nasal samples of the hospitalised group had a significantly higher proportion of microbes derived from surfaces, with a median of 0.21, than did those of the nonhospitalised group, with a median of 0.03 (*p* = 0.0016, determined using the Wilcoxon rank-sum test). This revealed that the nasal microbiota in hospitalised patients might be more susceptibly attributed from the surface microbiota, perhaps because of host factors that result in disequilibrium in nasal microbiota compared with those who are not hospitalised.

The proportions of abundant taxa in the nasal samples of the hospitalised and nonhospitalised groups are presented in Table 2 and Fig. S4. The analysis revealed that *Dysgonomonas* (*p* < 0.005), *Neisseria* (*p* = 0.019), and *Pseudomonas* (*p* = 0.045) were significantly more abundant in the hospitalised group than in the nonhospitalised group. *Corynebacterium* (*p* < 0.005) and *Moraxella* (*p* < 0.005) were more abundant in the nonhospitalised group. Additionally, *Streptococcus*, *Staphylococcus*, *Acinetobacter*, and unclassified genera into *Enterobacteriaceae* were more abundant in the hospitalised group, although the difference determined using the Wilcoxon rank-sum test was nonsignificant. These data imply that surface microbiota may correlate not only with nasal microbiota but also with the health status.

**Table 2:** The proportions and comparisons of abundant taxa of the nasal samples in hospitalised and nonhospitalised groups.

Taxonomy	Non-hospitalized group (A) Mean Proportion	Hospitalized group (B) Mean Proportion	B/A * 100	Wilcoxon test p-value
*Corynebacterium*	0.4581	0.2698	58.9	**<0.005**
*Dysgonomonas*	0.0062	0.0285	459.9	**<0.005**
*Staphylococcus*	0.0442	0.0923	208.6	0.7
*Streptococcus*	0.0443	0.0638	144.1	0.38
*Neisseria*	0.0165	0.0943	573.2	0.019
*Acinetobacter*	0.0022	0.019	881.5	0.9
unclassified genera into *Bacillales*	0.0024	0.0024	96.8	0.27
unclassified genera into *Enterobacteriaceae*	0.0191	0.0342	178.8	0.98
*Moraxella*	0.0805	0.0334	41.5	**<0.005**
*Pseudomonas*	0.0349	0.0462	132.2	0.045
*Alloiococcus*	0.0587	0.0083	14.2	**<0.005**
*Fusobacterium*	0.0067	0.0315	473.6	**<0.005**
*Propionibacterium*	0.0163	0.0049	30	**<0.005**
*Lactobacillus*	0.017	0.0112	65.7	0.76
*Streptophyta*	0.0088	0.0036	40.6	**<0.005**
unclassified genera into *Alcaligenaceae*	0.0333	0.0114	34.3	0.17
unclassified genera into *Xanthomonadaceae*	0.009	0.0119	132.1	0.71

## Discussion

Richly diverse commensal microbiota can provide protection to the host^1,11–14^, maintaining health by increasing resistance to infection^15^. Residents of HCIs and LTCFs are critical reservoirs for microorganisms, especially human-related microorganisms^23,26^. The bacterial fingerprints of surface microbiota represent a unique mix of bacterial communities found in various environments^27^. Lai *et al*.^9^ indicated that the environmental microbiome contributes to the composition of an individual’s nasal and skin microbiome, and the HCI environment may have had crucial implications for the health of the HCI residents in the current study. We determined the diversity and composition of environmental surface microbiota in three HCIs in this study and attempted to evaluate the relationship between surface and nasal microbiota. Six key discoveries were made. First, our data indicated that the microbiota distribution was different for the surface microbiota and nasal microbiota of participants between each HCI with different environmental building conditions. Second, the bacterial fingerprints of surface microbiota (from HCIs) and nasal microbiota (from HCI residents) overlapped. Third, 15 taxa were identified as being shared among all the nasal and surface samples. Nine of these core microbiota were human-related microorganisms, implying a strong risk of cross-infection at HCIs. Fourth, clinically significant human-related microorganisms—including *Streptococcus* spp., *Staphylococcus* spp., *Acinetobacter* spp., *Neisseria* spp., *Pseudomonas* spp., and unclassified genera into *Enterobacteriaceae—*were more abundant in the nasal communities of the hospitalised group compared with the nonhospitalised group. Fifth, the ventilation conditions of environment might influence the proportion of shared microbial communities between nasal and surface. Finally, the nasal microbiota of the hospitalised group may have been more highly correlated with the surface microbiota than those of the nonhospitalised group.

Our data obtained using 16S rRNA amplicon sequencing indicate much more diverse microbial communities in the three HCIs than was previously reported^7^. The dominant taxa in the surface microbial communities were *Corynebacterium* spp., *Dysgonomonas* spp., *Acinetobacter* spp., *Neisseria *spp., *Staphylococcus* spp., and unclassified genera into *Bacillales*, which is consistent with previous reports examining the bacterial environment on public restroom surfaces^28^, on gym surfaces^29^, in homes^9^, and on the human skin^27^. The dominant taxa in nasal microbial communities were C*orynebacterium* spp., *Neisseria* spp., *Staphylococcus* spp., and *Streptococcus* spp., which were reported to be associated with bronchiolitis^30^. Overall, the relative abundance of each genus differed by 6–56% between the three HCIs and 88 participants, indicating that human nasal and HCI community bacterial compositions are highly variable.

Further investigation of surface and nasal microbiota for the three HCIs and 88 participants revealed two community patterns: one at SC (located in a countryside community) and one at both of the other two HCIs (located in an urban community). SC is a new and rural LTCF, has open-window ventilation, and is located far from NH and NC. The variability of bacterial communities found on surfaces or in the nasal cavity may depend on the building and its location. Focusing on surface microbiota, the abundance of *Dysgonomonas* spp. ranged from 53.62% (NH) to 1.20% (SC), whereas the abundance of *Corynebacterium* ranged from 10.43% (SC) to 2.88% (NH). Conversely, focusing on nasal microbiota, the abundance of *Corynebacterium* spp. ranged from 48.60% (SC) to 21.53% (NH). Our previous study showed that the rate of nasal carriage for *A. baumannii* was approximately 54–92% at SC^23^ whereas that for* S. aureus* was approximately 0–15%. We suspect that most of the *Staphylococcus* spp. from SC were not *S. aureus* but other species, such as coagulase-negative *Staphylococcus*.

Several taxa of core microbiota, such as *Staphylococcus* spp., *Streptococcus* spp., *Acinetobacter* spp., unclassified genera into *Enterobacteriaceae*, *Pseudomonas* spp., *Propionibacterium* spp., *Proteus* spp., and unclassified genera into *Neisseriaceae*, are human-related microorganisms. Studies have previously identified these dominant human-related microorganisms and their putative routes of transmission, such as from the clothing of physicians and nursing staff^31^, stethoscopes^32^, personal phones^33^, and computer keyboards^34^. In the present study, four human-related microorganisms possibly belonging to the ‘ESKAPE’ group of organisms^35^ were discovered to be related to surface and nasal microbiota from the three HCIs. The high prevalence of human-related microorganisms in surface and nasal microbiota suggests that the current infection control strategies in these institutes may not be satisfactory, possibly because of a lack of staff or improper execution.

In agreement with a previous report^21^, the taxa of the identified core microbiota in this study, such as *Dysgonomonas* spp.*, Acinetobacter* spp.*, Staphylococcus* spp.*, Pseudomonas* spp.*, Corynebacterium* spp.*, Propionibacterium* spp., and unclassified genera into *Enterobacteriaceae*, were found to be associated with the human skin. Yano *et al*.^36^ demonstrated that a change in diversity within the hospital environmental microbiomes can result from hospital environmental contamination by medical staff pushing nursing wagons or sinks used by patients or visitors. According to Kleven *et al*.’s study, humans can be important dispersal vectors for microbes that colonise the built-up environment^37^. Whether *Dysgonomonas* spp. is a human pathogen remains controversial. *Corynebacterium* spp., *Dysgonomonas* spp., and *Moraxella* spp. were more abundant in the hospitalised participants than in the nonhospitalised participants in the present study. *Dysgonomonas* spp. was one of the most abundant and most prevalent taxa in the hospitalised group. *Dysgonomonas* is a gram-negative facultative anaerobic genus from the *Porphyromonadaceae* family and has been isolated from human sources^38^. *Corynebacterium* spp. was another of the most abundant and prevalent taxa in the hospitalised group. *Corynebacterium* species are commonly present in nature in soil, water, plants, and food products^39^. The non-diphtheroid *Corynebacterium* species can even be found in the mucosa and normal skin flora of humans and animals^39^.

In the present study, the nasal samples of the hospitalised group had a significantly higher proportion of microbes derived from surfaces than those of the nonhospitalised group (*p* = 0.0016), which indicates that compared with nonhospitalised patients. The nasal microbiota in hospitalised patients might be more susceptibly attributed from the surface microbiota because of host factors that result in disequilibrium in nasal microbiota^5–11^. Hospitalised patients may also have a less stable nasal bacterial composition and poorer health status^16^. Nasal microbiota compositions in the hospitalised group were more closely related than those in the nonhospitalised group to the microbial communities found in the surface environment. Lee *et al*. reported the importance of facing the existence of multiple-drug-resistant organisms in LTCFs^26^. Immune activation in mucosa and the resulting inflammation can exert selective pressures that favour some microorganisms but impair the fitness of most commensal microbes. For example, chronic rhinosinusitis can result from dysbiosis of nasal microbiota^20^. In addition, dysbiosis described in most cross-sectional studies is largely a consequence of diseases, such as is seen for inflammatory bowel disease (IBD)^40^. According to the study of Miyoshi *et al*.^38^, many of the components of IBD-associated microbiota are related to proinflammatory cytokines, which help sustain conditions that are favourable to microbiota while worsening chronic severe IBD. It remains unclear whether the dysbiosis of nasal microbiota is causative of, contributory to, or a consequence of hospitalisation. At present, identifying with certainty the causative microbial organisms is difficult because of limitations in technology and bioinformatics and difficulties in the clinical study design. Additionally, identifying the attribute directionality between the nasal microbiome and surface microbiome was difficult in the current study, and they could be in a bidirectional relationship. Studies should ideally be performed before the onset of the disease, which, in most cases, is impossible because who will develop a particular disease cannot be predicted accurately.

In conclusion, the results of the current study revealed an extremely high diversity within bacterial communities forming surface and nasal microbiota, and these two microbiota were found to differ. The bacterial fingerprints of the surface and nasal microbiota of individual HCIs and their residents overlapped, and nine of the 15 taxa from core microbiota are human-related microorganisms. Most surface and nasal microbiota can be considered nonpathogenic under normal circumstances, but there are potential risks in the three HCIs where residents are extremely prone to infections. In addition, the higher abundance of human-related microorganisms in the hospitalised group suggests the need for continuous monitoring of human-related microorganisms at HCIs, including LTCFs, to prevent healthcare-associated infections.

## Materials and Methods

### Ethics statement

This study was approved by the Ethics Committee of the Changhua Christian Hospital (CCH IRB No. 140318) to allow the collection of nasal samples. Each participant provided written informed consent under a protocol that was approved by the institutional review board, and all methods were performed in accordance with these guidelines.

### Nasal and environmental sample collection from three HCIs

Three HCIs, one LTCF referred to as SC with 49 beds in southern Nantou City, another LTCF to as NC with 49 beds in northern Nantou City, and one acute care hospital referred to as NH with 99 ward beds in a community hospital in northern Nantou City of central Taiwan (Fig. S5), were enrolled in this study. All participants were required to be residents of one of the HCIs during the study period. Participants were divided into hospitalised and nonhospitalised groups; those in the hospitalised group had been admitted to the acute care hospital within the 1 year prior to the study, whereas those in the nonhospitalised group had not been admitted within the 1 year prior to the study. All participants in the hospitalised group were hospitalized at Nantou Christian Hospital. Demographic characteristics of the participants in hospitalised group and nonhospitalised group were provide in Table S2.

All participants were required to be residents of one of the three HCIs (NH, NC, or SC) between January 1, 2014, and December 31, 2016. Eighty-eight participants were enrolled, 55 of whom comprised the hospitalised group and 33 of whom comprised the nonhospitalised group. The nasal cavity was sampled by inserting a swab into the nasal passage between the septum and middle turbinate, taking care to avoid contact with the nare wall, as was explained in the study conducted by Yan *et al*.^41^. The samples were transferred directly into Eppendorf tubes by using the provided swab and stored on ice and then at −20 °C until DNA isolation.

Eighty-three environmental samples from frequently touched areas in the patient room were collected two to four times by using a previously described protocol^5^. Environmental and infectious parameters for each HCI are summarised in Table S3. Sampling sites were divided into workplaces, frequently touched areas, and environments according to a previously described protocol^5^. All HCIs underwent terminal disinfection when a patient was discharged or their bed was changed, as described previously^5^. The air temperature, relative humidity, and mean CO_2_ concentration at sites with a central air conditioner (NH and NC) were slightly different from these conditions at the window-ventilated site (SC).

### 16S metagenomics sequencing analysis

Protocols for DNA extraction and PCR were described in our previous studies^5,42^. PCR amplification was performed using the V1 forward primer (5′-AGAGTTTGATCCTGGCTCAG-3′) and V2 reverse primer (5′-TGCTGCCTCCCGTAGGAGT-3′), producing a 349-bp amplicon spanning the highly variable V1-V2 region of the 16S rRNA gene sequence of the *E. coli* str. K12 substr. DH10B^43^. Paired-end sequence data in FASTQ format were obtained using the Illumina MiSeq platform, and a FASTX-Toolkit was used to assess sequence quality. The raw sequences of the nasal samples were deposited at the NCBI Sequence Read Archive (under bioproject accession number PRJNA437038). Raw reads were demultiplexed by barcodes, and adaptor sequences were removed. A minimum Phred quality score (Q score) of 20 was applied to trim low-quality bases. Reads with lengths of more than 150 nucleotides and joined paired-end reads were retained for further analysis.

### Microbial community analysis and statistical analysis

USEARCH^44^ was used to cluster remaining reads into OTUs by using a closed-reference OTU selection protocol at the 97% identity level against the Greengenes database^45^. QIIME^46^ was used to assess alpha diversity, beta diversity, and principal component analysis results obtained using the Bray-Curtis distance. The prevalence of a taxon was calculated by the number of samples containing the taxon divided the total number of samples, and the fisher’s test was used to determine significantly different prevalence between nasal and surface. The Wilcoxon rank-sum test was used to determine significant differences between the taxa from two groups of the samples. Taxa with a proportion of more than 0.1% in each sample were collected for the overlap analysis between the different groups of samples. OTUs that were present in more than 90% of all nasal samples were collected as nasal abundant microbiota, and SourceTracker was used to identify the contribution of environmental microbiota to nasal microbiota.

### Accession codes

The raw sequences were deposited at the NCBI sequence Read Archive under the Bioproject accession number PRJNA437038.

## Supplementary information

Supplementary Dataset 1
